# Supplementary material for: Cardiometabolic outcomes up to 12 months after COVID-19 infection. A matched cohort study in the UK
Source: PLoS Med. 2022 Jul 19;19(7):e1004052. doi: 10.1371/journal.pmed.1004052 (PMC9295991; doi:10.1371/journal.pmed.1004052)
Supplement: S1 Protocol — (PDF) [file pmed.1004052.s002.pdf]

1

General information

Protocol reference Id
20_000265

Study title
Cohort study of risk factors of acute and chronic cardiovascular events in COVID-19 patients

Research Area

Disease Epidemiology

Does this protocol describe an observational study using purely CPRD data?
Yes

Does this protocol involve requesting any additional information from GPs, or contact with patients?
No

2

Research team

**Role**	Chief Investigator
**Title**	Professor of Public Health
**Full name**	Martin Gulliford
**Affiliation/organisation**	King's College London (KCL)
**Email**	martin.gulliford@kcl.ac.uk
**Will this person be analysing the data?**	Yes
**Status**	Confirmed

**Role**	Corresponding Applicant
**Title**	Research associate
**Full name**	Emma Rezel-Potts
**Affiliation/organisation**	King's College London (KCL)
**Email**	emma.rezel-potts@kcl.ac.uk
**Will this person be analysing the data?**	Yes
**Status**	Confirmed

3

Access to data

Sponsor
King's College London (KCL)

Funding source for the study
Is the funding source for the study the same as Chief Investigator's affiliation?
Yes
Funding source for the study
King's College London (KCL)

Institution conducting the research
Is the institution conducting the research the same as Chief Investigator's affiliation?
Yes
Institution conducting the research
King's College London (KCL)

Method to access the data
Indicate the method that will be used to access the data
Institutional multi-study licence
Is the institution the same as Chief Investigator's affiliation?
Yes
Institution name
King's College London (KCL)

Extraction by CPRD
Will the dataset be extracted by CPRD
No

Multiple data delivery
This study requires multiple data extractions over its lifespan
No
Number of repeated data extractions required over the lifespan of this study

Data processors

**Data processor is**	Same as the chief investigator's affiliation
**Processing**	Yes
**Accessing**	Yes
**Storing**	Yes
**Processing area**	UK

4

Information on data

Primary care data

CPRD GOLD

CPRD Aurum

Do you require data linkages

Yes

Patient level data

HES Admitted Patient Care

ONS Death Registration Data

NCRAS data

Covid 19 linkages

Area level data

Do you require area level data?

Yes

Practice level (UK)

Practice Level Index of Multiple Deprivation

Patient level (England only)

Patient Level Index of Multiple Deprivation

Withheld concepts

Are withheld concepts required?

No

Linkage to a dataset not listed

Are you requesting a linkage to a dataset not listed?

No

Patient data privacy

Does any person named in this application already have access to any of these data in a patient identifiable form, or associated with an identifiable patient index?

No

5

Protocol information

Lay Summary

This research aims to inform patient management during the present COVID-19 coronavirus pandemic. Early findings suggest that patients with COVID-19 commonly experience conditions affecting the heart and blood vessels (cardiovascular conditions) such as heart failure. Acute heart failure often leads to very severe outcomes including death. We are also interested in understanding the risk factors for longer-term cardiovascular outcomes in COVID-19 patients such as chronic heart failure. Those with cardiovascular risk factors, such as high blood pressure, and who have experienced cardiovascular conditions in the past, seem to be at increased risk of poor COVID-19 outcomes. However, studies have reported several cases of cardiovascular events following a COVID-19 diagnosis in patients without a history of cardiovascular disease. It appears that the SARS-CoV-2 virus that causes COVID-19, might be capable of directly causing damage to the heart and more research is required to understand which patients are likely to benefit from dedicated, specialist cardiac care and monitoring. This study will use Clinical Practice Research Datalink (CPRD) data to collate cardiovascular risk factors in COVID-19 patients to assess their association with cardiovascular events including acute heart failure, chronic heart failure and death. We will analyse data from patients who have diagnoses of heart failure or other cardiovascular conditions, comparing risk factors and disease progression among those with a preceding COVID-19 diagnosis to those without.

Technical Summary

Cardiovascular risk factors and heart conditions have been associated with increased risk of poor COVID-19 outcomes. Several hospitalised COVID-19 patients have developed cardiovascular outcomes including acute heart failure, chronic heart failure, pulmonary edema and myocardial infarction. Pre-existing cardiovascular disease seems to be an important driver of COVID-19 severity, however other studies have noted serious cardiovascular outcomes in COVID-19 patients without a history of cardiovascular disease. SARS-CoV-2 could directly induce myocardial damage, potentially via the proinflammatory cytokine storm. We seek to improve evidence in this area for earlier identification of COVID-19 patients at high risk of cardiovascular outcomes who could benefit from dedicated cardiac monitoring and early referral to specialist teams. We will use the Clinical Practice Research Datalink (CPRD) Aurum and GOLD datasets to conduct a population cohort study, evaluating outcomes among patients with a COVID-19 diagnosis compared to a matched cohort without a COVID-19 diagnosis. Outcomes will be hospital readmission, mortality, diabetes events and cardiovascular events following COVID-19 diagnosis. Cox proportional hazards regression models will use hazards ratios to compare outcomes to a cohort of patients matched to the COVID-19 patients on practice, gender and year of birth and no COVID-19 diagnosis during the study period. Matched analyses will be adjusted for covariates including ethnicity; risk profiles between groups will be compared. Cardiovascular disease risk factors will be compiled including smoking status, blood pressure, cholesterol, diabetes and body mass index. Hospital admissions and cardiovascular events will be identified using the Hospital Episode Statistics Admitted Patient Care registry. We will use the Office for National Statistics (ONS) Death Registration Data for mortality data if possible, but note that the latest release (set 20) only covers up to June 2020. Should there be no further release prior to initiating our analysis, we will use the CPRD Aurum and GOLD death dates instead. A secondary analysis will evaluate longer-term cardiovascular effects of COVID-19 using CPRD data on relevant prescriptions including diuretics and anticoagulants.

Outcomes to be measured

Primary analysis:
- Hospital admission.
- Mortality.
- Diabetes.
- Cardiovascular disease diagnoses.

Outcomes will be evaluated over the duration of the study period.

Secondary analysis
Chronic cardiovascular conditions following index dates among those surviving, measured according to prescriptions for:
- diuretics
- anticoagulants
- aspirin
- antihypertensive drugs
- antiplatelet drugs
- lipid lowering drugs (mainly statins).

Objectives, specific aims & rationale

Evidence is limited with some studies providing conflicting evidence as to the extent to which history of cardiovascular disease influences the severity of COVID-19 outcomes. Greater understanding of who in the population may be at increased risk of severe cardiovascular events or mortality following COVID-19 diagnosis may help to inform patient management. We aim to evaluate the effects of COVID-19 on severity of cardiovascular outcomes and mortality. We will examine associations between COVID-19 diagnosis and mortality, cardiovascular diagnoses, hospital admissions and diabetes, comparing a cohort with a COVID-19 diagnosis to a matched cohort without a COVID-19 diagnosis. We also aim to compare the cardiovascular risk profiles of both cohorts. A secondary objective is to evaluate longer-term cardiovascular effects of COVID-19 using CPRD data on prescriptions for diuretics, anticoagulants, antiplatelets, antihypertensives, statins and aspirin.

Study background

Coronavirus disease 2019 (COVID-19) caused by the novel severe acute respiratory syndrome coronavirus 2 (SARS-CoV-2) has become a global pandemic causing substantial morbidity and mortality. A growing body of research characterises COVID-19 as a multi-systemic condition which frequently has cardiac manifestations (1-6). Early studies reported heart failure as among the most frequent COVID-19 complications, observed in as many as 24% of all patients and in half of patients who died (7,8).

There have been various postulated mechanisms for cardiovascular outcomes following COVID-19. SARS-CoV-2 enters host cells via interaction with the angiotensin-converting enzyme 2 (ACE2) receptor which is part of the renin-angiotensin-aldosterone system (RAAS). ACE2 receptors are abundant in the cardiovascular system so it is suggested that SARS-CoV-2 is internalised into cardiovascular cells via this route, resulting in acute cardiac injury (ACI). The proinflammatory cytokine storm is a mechanism which could incite a dysregulated immune response and plaque rupture leading to acute coronary syndrome (ACS) (9,10). ACS, in particular type 2 myocardial infarction (MI) could also be the result of hypoxia causing an oxygen supply–demand mismatch (9). Left ventricular dysfunction/heart failure is thought to be due to microvascular thrombi formation and intravascular coagulation or a dysregulated immune response that surges after viral response. Direct cardiotropic myocardial injury could be induced by oxidative stress triggered by the virus. Finally, SARS-CoV-2–induced ACE-mediated damage whereby SARS-CoV-2 causes hyperstimulation leading to responses including vasoconstriction, inflammation and fibrosis (5). This same process can have inhibitory effects causing vasodilation and anti-fibrosis. It had been suggested that RAAS-inhibitors commonly used to treat conditions including hypertension and diabetes may enhance ACE2 activity and thereby COVID-19 susceptibility and severity but mounting evidence, including from our own research team using the CPRD, has not supported this (12).

Patients with cardiovascular disease who develop COVID-19 have a higher risk of mortality (13-14). Risk factors such as hypertension, high body mass index (BMI), older age and comorbidities like diabetes mellitus have also been associated with increased severity of COVID-19 disease outcomes (15). Among COVID-19 patients who develop ACI, approximately 30% and 60% had a history of coronary heart disease and hypertension, respectively (16). Despite the growing body of literature in the short time period since COVID-19 first emerged, there remains much to understand about coronavirus disease and risk factors of cardiovascular outcomes in COVID-19 patients. A study of patients presenting with COVID-19 in emergency care in Madrid, Spain, found that among those diagnosed with acute heart failure, the majority (78%) developed in patients without a history of heart failure (17). Another study of hospitalised patients in London, UK, found that pre-existing cardiovascular disease to be a key trigger of COVID-19 mortality (18). It may be that the direct effects of SARS-CoV-2 on the cardiovascular system means more inclusivity is required when determining which patients may be at greatest risk of cardiovascular events. We therefore aim to use the CPRD to identify a cohort of COVID-19 patients experiencing cardiovascular events including including acute heart failure and myocardial infarction, comparing outcomes of mortality and hospital admission to a matched cohort with similar cardiovascular events but no COVID-19 diagnosis. We seek to improve knowledge in this area for earlier identification of COVID-19 patients at high risk of cardiovascular events who could benefit from more dedicated cardiac monitoring and early referral to specialist teams.

Study type

Hypothesis testing

Study design

Population cohort study

Feasibility counts

In the January 2021 release of CPRD there are 309,612 unique patients in CPRD GOLD with Covid codes and 2,140,328 in CPRD Aurum. In CPRD Gold, there are 8,459 patients with a confirmed diagnosis and 26,886 with a possible diagnosis. In CPRD Aurum, there are 248,307 patients with a confirmed diagnosis and 154,117 with a possible diagnosis.

These may be contrasted with figures at the end of the first wave in the July release of CPRD when there were 2,310 CPRD Gold patients with a confirmed diagnosis and 13,795 with a possible diagnosis, as well as 27,113 CPRD Aurum patients with a confirmed diagnosis and 93,039 with a possible diagnosis.

If we include patients with possible or confirmed diagnoses in CPRD Aurum, there will be a total of 402,424 patients on whom 62% have confirmed diagnoses. However, among 120,152 first wave patients in CPRD Aurum, only 23% have a confirmed diagnosis. Therefore, we plan to include wave (first or second) and diagnosis confirmation as covariates, as well as exploring possible effect modification in relation to wave and diagnosis confirmation.

Sample size considerations

The COVID-19 case fatality rate has been estimated to be between 1.0 and 3.4% (19) and hospital readmissions at about 20% (20), depending on the time of the pandemic. With a total sample of 402,424 patients, there may be about 80,000 requiring hospitalisation and 16,000 experiencing cardiovascular events. If there are 16,000 patients with COVID-19 experiencing cardiovascular outcomes and 3,200 are readmitted to hospital after discharge, there will be 90% power to detect hazard ratios of 1.12. Under plausible scenarios very small relative risks will be detectable, the main problem therefore will be control of bias, rather than lack of precision.

Planned use of linked data and benefit to patients in England and Wales

We plan to use linked HES APC data to determine cardiovascular outcomes in hospitalised COVID-19 patients. This will increase our understanding of the pathogenesis and treatment of severe COVID-19 helping to meet our overall aim to to improve evidence for earlier identification of COVID-19 patients at high risk of cardiovascular outcomes who could benefit from dedicated cardiac monitoring and early referral to specialist teams.

To obtain measures of deprivation data for use as confounders, we will also use practice- and patient-level Index of Multiple Deprivation data. We will use ONS Death Registration Data for mortality data if possible, but note that the latest release (set 20) only covers up to June 2020. Should there be no further release prior to initiating our analysis, we will use the CPRD Aurum and GOLD death dates instead.

Definition of the study population

FF. The study recruitment period will be from 29th January 2020 (the date of the first UK COVID-19 case) to present. The study population will be the cohort of registered patients in CPRD Aurum. We will include acceptable patients with defined gender (Gender=1 or 2). We will include patients with a confirmed or suspected COVID-19 diagnosis in CPRD Aurum using code lists for COVID-19 (Appendix 1). This will be compared to a cohort matched on practice, gender and year of birth with no COVID-19 diagnosis during the study period. The index date will be the first COVID-19 diagnosis. The start date for each patient will be the patient current registration date. The end date will be the earliest of the patient end of registration, patient death date, or last data collection date. In order to identify incident events, we will take the patient start date to be no earlier than 6 months after the current registration date. We will validate outcomes using the CPRD GOLD, checking that coefficients are of similar magnitude across the two databases. In the CPRD GOLD, the patient start date will be the later of the patient current registration date or the practice UTS date and the end date will be determined using the same approach as for Aurum.

Selection of comparison groups/controls

Cohort study: the cohort will comprise all those with confirmed or suspected COVID-19.

Comparison group: a matched cohort of patients who not been diagnosed with COVID-19 during the study period. Matching will be on the basis of practice, year of birth and gender.

Exposures, outcomes and covariates

Exposures:
1) Clinical diagnoses of COVID-19 determined using CPRD Aurum.

Outcomes:
Cardiovascular conditions as follows:
Acute cardiac injury following index date:
- Left ventricular dysfunction/heart failure
- Acute heart failure with reduced and preserved ejection fraction (HFrEF/HFpEF), cardiogenic shock
- Myocardial injury
- Acute myocarditis
- Arrhythmias
- Stress-induced cardiomyopathy
- Acute pericarditis
- Thromboembolic complications.
- Acute coronary syndrome (ACS) including:
o myocardial infarction (MI) type 1 and type 2
o non-ST-elevation MI (NSTEMI)
o unstable angina

Hospital admission following COVID-19 diagnosis (or readmission if originally diagnosed in hospital).
Mortality from the CPRD GOLD/Aurum death date. We will use the ONS Death Registration Data for mortality data if possible, but note that the latest release (set 20) only covers up to June 2020 so may need to use the CPRD Aurum death dates instead. We will evaluate outcomes across the entirety of the study period and will also conduct sensitivity analyses to compare outcomes at 28 days, as this is the Public Health England timeframe for COVID-19 deaths, and 120 days, which will cover the longer-term effects of Long COVID.

Covariates:
Known risk factors for cardiovascular disease: smoking status; BMI; blood pressure; cholesterol; comorbidities including diabetes. Previous diagnoses of cardiovascular conditions. See section of confounding for further risk factors of interest.
Risk factors will be determined using CPRD Aurum.

Data/statistical analysis

The CPRD Aurum will be used to identify a cohort of COVID-19 patients who experience subsequent cardiovascular events and a matched cohort experiencing similar events but without COVID-19. Outcomes will be hospital admission and mortality within 60 days. Cardiovascular disease risk factors will be compiled including smoking status, blood pressure and body mass index. Cox proportional hazards regression models will test associations between COVID-19 diagnosis and outcomes, adjusting for important covariates including ethnicity, gender, frailty and deprivation measures. We will evaluate effect modification by age-group and comorbidity status. We will compare hazard ratios according to cohort risk factors. We will use robust estimators to allow for clustering by practice/matched set, though this is likely to have minimal effect. We will evaluate the proportional hazards assumption using the Schoenfeld residuals. We will validate outcomes using the CPRD GOLD, checking that coefficients are of similar magnitude across the two databases.

At the reporting stage, we will follow ONS rules on low cell frequencies, rounding frequencies between 0 and 5 to either 0 or 5 (with an explanatory note) in order to avoid any possible deductive disclosure.

Plan for addressing confounding

We will adjust for age and sex, as well as clustering by general practice. In addition, we will adjust for index of multiple deprivation quintiles and ethnicity.

We will adjust for co-existing health conditions (comorbidities).

We will also employ Clegg's e-Frailty index which evaluates 36 conditions and develops a frailty score based on the number of conditions diagnosed in a patient. We will use the Read code lists provided by the e-Frailty Index developers (21).

Plans for addressing missing data

Missing data in electronic health records are typically missing not at random, because GPs record information when there is a reason to do so. Consequently, multiple imputation methods generally have limited application.

A main concern may be that data close to the end date may not be complete if the last data collection date is before an outcome is recorded. We can evaluate this by conducting a sensitivity analysis, using only data that were recorded more than 15 or more than 30 days before the last collection date. The sensitivity analysis will have reduced power but we can evaluate whether there may be any important changes in estimates as compared to the analysis with all recorded data.

Patient or user group involvement

None to date but we can obtain PPI input from existing patient groups from the NIHR Biomedical Research Centre at Guy's and St Thomas' Hospitals.

Plans for disseminating & communicating

We will submit our results to regulatory and professional bodies and publish in peer-review journals.

Conflict of interest statement

We report no conflicts of interest.

Limitations of study design

A study like this has many limitations, so we will need to be very careful interpreting our results. Most patients with COVID-19 are advised to self-isolate at home without contacting their GP. Consequently, those recorded in general practice represent a selected sample, which might include a higher proportion with co-existing conditions like hypertension. Also, as we will be analysing recently recorded data, this may yet be incomplete for key outcomes. A central limitation will be the possible under-reporting of acute cardiac injury including heart failure in COVID-19 patients. Particularly in the initial stages of the pandemic, the multi-system manifestations of the disease may not have been well understood. Overwhelmed hospitals and protocol around infection control may have also hampered accurate cardiac monitoring and reporting of causes of morbidity and mortality. It is also likely that some in the matched cohort will have had COVID-19 but not been diagnosed or diagnoses may not have been recorded due to attendance at an alternative provider.

References

1) Basu-Ray I, Almaddah Nk, Adeboye A, et al. Cardiac Manifestations Of Coronavirus. [Updated 2020 Aug 8]. In: StatPearls [Internet]. Treasure Island (FL): StatPearls Publishing; 2020 Jan-. Available from: https://www.ncbi.nlm.nih.gov/books/NBK556152/

2) Hendren NS, Drazner MH, Bozkurt B, Cooper LT, Jr. Description and Proposed Management of the Acute COVID-19 Cardiovascular Syndrome. Circulation. 2020;141(23):1903-14. doi:10.1161/circulationaha.120.047349.

3) Inciardi RM, Lupi L, Zaccone G, Italia L, Raffo M, Tomasoni D et al. Cardiac Involvement in a Patient With Coronavirus Disease 2019 (COVID-19). JAMA Cardiology. 2020;5(7):819-24. doi:10.1001/jamacardio.2020.1096.

4) Aghagoli, G, Gallo Marin, B, Soliman, LB, Sellke, FW. Cardiac involvement in COVID19 patients: Risk factors, predictors, and complications: A review. J Card Surg. 2020; 35: 1302–1305. doi:10.1111/jocs.14538.

5) Shaha KB, Manandhar DN, Cho JR, et alCOVID-19 and the heart: what we have learnt so farPostgraduate Medical Journal Published Online First: 17 September 2020. doi: 10.1136/postgradmedj-2020-138284.

6) Samuel S, Friedman RA, Sharma C, Ganigara M, Mitchell E, Schleien C et al. Incidence of arrhythmias and electrocardiographic abnormalities in symptomatic pediatric patients with PCR-positive SARS-CoV-2 infection, including drug-induced changes in the corrected QT interval. Heart rhythm. 2020;17(11):1960-6. doi:10.1016/j.hrthm.2020.06.033.

7) Chen, T. et al. Clinical characteristics of 113 deceased patients with coronavirus disease 2019: retrospective study. BMJ 368, m1091 (2020).

8) Zhou, F. et al. Clinical course and risk factors for mortality of adult inpatients with COVID-19 in Wuhan, China: a retrospective cohort study. Lancet 395, 1054–1062 (2020).

9) Tersalvi G, Vicenzi M, Calabretta D, Biasco L, Pedrazzini G, Winterton D. Elevated Troponin in Patients With Coronavirus Disease 2019: Possible Mechanisms. Journal of cardiac failure. 2020;26(6):470-5. doi:https://doi.org/10.1016/j.cardfail.2020.04.009.

10) Rabi, F.A.; Al Zoubi, M.S.; Kasasbeh, G.A.; Salameh, D.M.; Al-Nasser, A.D. SARS-CoV-2 and Coronavirus Disease 2019: What We Know So Far. Pathogens 2020, 9, 231.

11) Tomasoni D, Inciardi RM, Lombardi CM, et al. Impact of heart failure on the clinical course and outcomes of patients hospitalized for COVID-19. Results of the Cardio-COVID-Italy multicentre study. European Journal of Heart Failure 2020;n/a(n/a) doi: https://doi.org/10.1002/ejhf.2052.

12) Rezel-Potts E, Douiri A, Chowienczyk P, et al. Antihypertensive Medications and COVID-19 Diagnosis and Mortality: Population-based Case-Control Analysis in the United Kingdom. medRxiv 2020.09.25.20201731 doi: https://doi.org/10.1101/2020.09.25.20201731.

13) Ssentongo P, Ssentongo AE, Heilbrunn ES, Ba DM, Chinchilli VM. Association of cardiovascular disease and 10 other pre-existing comorbidities with COVID-19 mortality: A systematic review and meta-analysis. PloS one. 2020;15(8):e0238215. doi:10.1371/journal.pone.0238215.

14) Pranata R, Huang I, Lim MA, Wahjoepramono EJ, July J. Impact of cerebrovascular and cardiovascular diseases on mortality and severity of COVID-19–systematic review, meta-analysis, and meta-regression. Journal of Stroke and Cerebrovascular Diseases. 2020:104949.

15) Williamson EJ, Walker AJ, Bhaskaran K, Bacon S, Bates C, Morton CE et al. Factors associated with COVID-19-related death using OpenSAFELY. Nature. 2020;584(7821):430-6. doi:10.1038/s41586-020-2521-4.
16) Lippi G, Lavie CJ, Sanchis-Gomar F. Cardiac troponin I in patients with coronavirus disease 2019 (COVID-19): Evidence from a meta-analysis. Progress in cardiovascular diseases. 2020;63(3):390-1. doi:10.1016/j.pcad.2020.03.001.
17) Rey JR, Caro-Codón J, Rosillo SO, et al. Heart failure in COVID-19 patients: prevalence, incidence and prognostic implications. European Journal of Heart Failure 2020;n/a(n/a) doi: https://doi.org/10.1002/ejhf.1990.
18) O'Gallagher K, Shek A, Bean DM, Bendayan R, Teo JTH, Dobson RJB et al. Pre-existing cardiovascular disease rather than cardiovascular risk factors drives mortality in COVID-19. medRxiv. 2020:2020.12.02.20242933. doi:10.1101/2020.12.02.20242933.
19) John Hopkins University & Medicine. Coronavirus Resource Center. Mortality Analyses. https://coronavirus.jhu.edu/data/mortality (Accessed 17th December 2020).
20) Donnelly JP, Wang XQ, Iwashyna TJ, Prescott HC. Readmission and Death After Initial Hospital Discharge Among Patients With COVID-19 in a Large Multihospital System. JAMA. Published online December 14, 2020. doi:10.1001/jama.2020.21465

21) Clegg A, Bates C, Young J, Ryan R, Nichols L, Ann Teale E, Mohammed MA, Parry J, Marshall T. Development and validation of an electronic frailty index using routine primary care electronic health record data. Age Ageing. 2016 May;45(3):353-60. doi: 10.1093/ageing/afw039.

Appendices

chd_medcodeids_snomed_0.txt

covidconceptids.txt

Grant ID
