# Supplementary material for: Cardiometabolic outcomes up to 12 months after COVID-19 infection. A matched cohort study in the UK
Source: PLoS Med. 2022 Jul 19;19(7):e1004052. doi: 10.1371/journal.pmed.1004052 (PMC9295991; doi:10.1371/journal.pmed.1004052)
Supplement: S1 Text — (DOCX) [file pmed.1004052.s004.docx]

431,193 controls without prevalent CVD or DM matched on gender, year of birth and general practice

2,543 matched sets excluded:

- prevalent CVD (961) or DM (123) identified from full medical record
- indeterminate gender (32)
- age > 104 years (1,427)

14,911,765 patients registered in March 2021 release of CPRD Aurum

428,650 matched sets included in analysis

85,792 with prevalent CVD or DM

431,193 participants diagnosed with Covid-19

516,985 case participants diagnosed with Covid-19 after 29 January 2020
