# Supplementary material for: Cardiometabolic outcomes up to 12 months after COVID-19 infection. A matched cohort study in the UK
Source: PLoS Med. 2022 Jul 19;19(7):e1004052. doi: 10.1371/journal.pmed.1004052 (PMC9295991; doi:10.1371/journal.pmed.1004052)
Supplement: S2 Text — (DOCX) [file pmed.1004052.s005.docx]

AdjustedModel<-summary(geese(IncCVD~phase+Case+phase*Case+I(age)+I(age^2)+ gender+

Ethnicity+smokstat+BMIcat+SBPcat+CharlsonScore+I(indexmonth)+I(indexmonth^2)+

offset(log(pw)),id=set,corstr="exch",family=poisson(),data=results))

UnadjustedModel<-summary(geese(IncCVD~phase+Case+phase*Case+I(indexmonth)+

I(indexmonth^2)+offset(log(pw)),id=set,corstr="exch",family=poisson(),data=results))

Phase is a factor representing the periods ‘pre-index’, zero to four weeks’ ‘post-acute’, five to 12 weeks; and ‘long’ 13 to 52 weeks

Case: 0=control; 1=Covid-19
