# Supplementary material for: Cardiometabolic outcomes up to 12 months after COVID-19 infection. A matched cohort study in the UK
Source: PLoS Med. 2022 Jul 19;19(7):e1004052. doi: 10.1371/journal.pmed.1004052 (PMC9295991; doi:10.1371/journal.pmed.1004052)
Supplement: S3 Text — (DOCX) [file pmed.1004052.s006.docx]

| **Medical Term** | **Frequency** |
| --- | --- |
|  |  |
| SARS-CoV-2 RNA (ribonucleic acid) detection result positive | 279,783 |
| COVID-19 confirmed by laboratory test | 19,008 |
| SARS-CoV-2 detection result positive | 18,564 |
| COVID-19 | 6,640 |
| SARS-CoV-2 IgG detection result positive | 5,679 |
| SARS-CoV-2 antigen detection result positive | 2,880 |
| COVID-19 | 2,798 |
| Coronavirus infection | 2,362 |
| Disease caused by 2019-nCoV | 1,633 |
| Disease caused by 2019-nCoV | 1,402 |
| COVID-19 severity score | 1,035 |
| Detection of 2019-nCoV using polymerase chain reaction technique | 988 |
| Detection of SARS-CoV-2 using polymerase chain reaction technique | 715 |
| Confirmed 2019-nCoV infection | 672 |
| COVID-19 confirmed using clinical diagnostic criteria | 651 |
| SARS-CoV-2 detected | 451 |
| Assessment using COVID-19 severity scale | 424 |
| SARS-CoV-2 IgA detection result positive | 306 |
| SARS-CoV-2 antibody detection result positive | 299 |
| Pneumonia caused by SARS-CoV-2 | 192 |
| COVID-19 severity scale | 131 |
| SARS-CoV-2 IgM detection result positive | 110 |
| COVID-19 confirmed clinically | 71 |
| Pneumonia caused by SARS-CoV-2 | 49 |
| Possible COVID-19 | 49 |
| Pneumonia caused by 2019-nCoV | 19 |
| COVID-19 pneumonia | 18 |
| Pneumonia caused by 2019-nCoV | 16 |
| Probable COVID-19 confirmed using clinical diagnostic criteria | 13 |
| Other codes (14 codes) | 53 |
|  |  |
| **Suspected Covid-19** |  |
| Suspected COVID-19 | 33,356 |
| Telephone consultation for suspected 2019-nCoV | 18,449 |
| Suspected disease caused by 2019-nCoV | 15,043 |
| Suspected coronavirus infection | 10,537 |
| Suspected 2019-nCoV infection | 3,540 |
| Telephone consultation for suspected SARS-CoV-2 | 597 |
| Suspected disease caused by Wuhan 2019-nCoV | 107 |
| Other codes (3 codes) | 10 |
|  |  |
