# Supplementary material for: Cardiometabolic outcomes up to 12 months after COVID-19 infection. A matched cohort study in the UK
Source: PLoS Med. 2022 Jul 19;19(7):e1004052. doi: 10.1371/journal.pmed.1004052 (PMC9295991; doi:10.1371/journal.pmed.1004052)
Supplement: S4 Text — CI, confidence interval; RR, adjusted incidence rate ratio. (Estimates were adjusted for age, ethnicity, smoking, BMI category, SBP category, Charlson score, index month and matched set.) (DOCX) [file pmed.1004052.s007.docx]

|  | **Incidence of CVD** | | **Incidence of DM** |  |
| --- | --- | --- | --- | --- |
|  | **RR (95% CI)** | **P value** | **RR (95% CI)** | **P value** |
|  |  |  |  |  |
| **Net effect of Covid-19^a,b^** |  |  |  |  |
| Acute Covid-19 (up to 4 weeks from index) | 5.82 (4.82 to 7.03) | <0.001 | 1.81 (1.51 to 2.19) | <0.001 |
| Post-acute Covid-19 (5-12 weeks from index) | 1.49 (1.28 to 1.73) | <0.001 | 1.27 (1.11 to 1.46) | <0.001 |
| Long Covid-19 (13-52 weeks from index) | 0.80 (0.73 to 0.88) | <0.001 | 1.07 (0.99 to 1.16) | 0.07 |
|  |  |  |  |  |
| **Controls^c^** |  |  |  |  |
| Before index date | Ref. |  | Ref. |  |
| 4 weeks from index date | 0.98 (0.82 to 1.17) | 0.80 | 0.84 (0.72 to 0.99) | 0.04 |
| 5-12 weeks from index date | 1.12 (0.99 to 1.28) | 0.07 | 0.99 (0.89 to 1.10) | 0.85 |
| 13-52 weeks from index date | 1.21 (1.13 to 1.30) | <0.001 | 1.17 (1.10 to 1.24) | <0.001 |
|  |  |  |  |  |
| **Overall difference between Covid-19 patients and controls^d^** | |  |  |  |
| Control | Ref. |  | Ref. |  |
| Covid-19 patients | 1.73 (1.63 to 1.84) | <0.001 | 1.18 (1.12 to 1.25) | <0.001 |
|  |  |  |  |  |

^a^group by time interaction; ^b^ additional effect, net of rate in pre-index period for cases and rate in controls in same period; ^c^ ‘time’ effect; ^d^ ‘group’ effect
