# Supplementary material for: Cardiometabolic outcomes up to 12 months after COVID-19 infection. A matched cohort study in the UK
Source: PLoS Med. 2022 Jul 19;19(7):e1004052. doi: 10.1371/journal.pmed.1004052 (PMC9295991; doi:10.1371/journal.pmed.1004052)
Supplement: S5 Text — Adjusted rate ratios (95% confidence intervals) by 4-week period following COVID-19. (Estimates were adjusted for age, ethnicity, smoking, body mass index category, systolic blood pressure category, Charlson score, index month, and matched set.) (DOCX) [file pmed.1004052.s008.docx]

| **Weeks from Covid-19 onset** | | **Cardiovascular Events** | | | **Diabetes Mellitus** | | | |
| --- | --- | --- | --- | --- | --- | --- | --- | --- |
|  | **RR** | **Lower 95% limit** | **Upper 95% limit** | **P value** | **RR** | **Lower 95% limit** | **Upper 95% limit** | **P value** |
|  |  |  |  |  |  |  |  |  |
| 0 to 3 | 5.83 | 4.82 | 7.04 | 0.000 | 1.82 | 1.51 | 2.19 | 0.000 |
| 4 to 7 | 1.84 | 1.51 | 2.24 | 0.000 | 1.36 | 1.13 | 1.65 | 0.001 |
| 8 to 11 | 1.15 | 0.94 | 1.42 | 0.18 | 1.20 | 1.00 | 1.43 | 0.06 |
| 12 to 15 | 0.88 | 0.72 | 1.08 | 0.21 | 1.14 | 0.96 | 1.36 | 0.14 |
| 16 to 19 | 0.85 | 0.69 | 1.04 | 0.12 | 1.24 | 1.04 | 1.47 | 0.02 |
| 20 to 23 | 0.74 | 0.60 | 0.91 | 0.005 | 1.29 | 1.08 | 1.55 | 0.005 |
| 24 to 27 | 0.73 | 0.59 | 0.90 | 0.003 | 1.03 | 0.86 | 1.22 | 0.78 |
| 28 to 31 | 0.92 | 0.74 | 1.15 | 0.48 | 1.03 | 0.87 | 1.23 | 0.71 |
| 32 to 35 | 0.84 | 0.66 | 1.05 | 0.13 | 1.02 | 0.85 | 1.23 | 0.84 |
| 36 to 39 | 0.69 | 0.55 | 0.85 | 0.001 | 1.01 | 0.84 | 1.21 | 0.95 |
| 40 to 43 | 0.91 | 0.73 | 1.14 | 0.41 | 0.97 | 0.80 | 1.17 | 0.75 |
| 44 to 27 | 0.87 | 0.69 | 1.09 | 0.23 | 0.98 | 0.82 | 1.17 | 0.81 |
| 48 to 51 | 0.66 | 0.51 | 0.84 | 0.001 | 1.09 | 0.90 | 1.32 | 0.38 |
|  |  |  |  |  |  |  |  |  |
