# Supplementary material for: Cardiometabolic outcomes up to 12 months after COVID-19 infection. A matched cohort study in the UK
Source: PLoS Med. 2022 Jul 19;19(7):e1004052. doi: 10.1371/journal.pmed.1004052 (PMC9295991; doi:10.1371/journal.pmed.1004052)
Supplement: S6 Text — (DOCX) [file pmed.1004052.s009.docx]

|  |  | **Pre-Index** | **Acute** | **Post-Acute** | **Long** |
| --- | --- | --- | --- | --- | --- |
|  |  |  |  |  |  |
| **Incident diabetes mellitus patients (13,063)** | | |  |  |  |
|  |  |  |  |  |  |
| Number | Covid-19 | 3,461 | 420 | 681 | 3,256 |
|  | Controls | 2,542 | 166 | 384 | 2,542 |
|  |  |  |  |  |  |
| Age (median, IQR, years) | Covid-19 | 47 (35 to 57) | 50 (38 to 59) | 47 (34 to 56) | 46 (34 to 56) |
|  | Controls | 46 (33 to 57) | 45 (30 to 56) | 43 (32 to 55) | 45 (33 to 56) |
|  |  |  |  |  |  |
| Age≤35 years | Covid-19 | 924 (27) | 83 (20) | 188 (28) | 958 (29) |
|  | Controls | 747 (29) | 55 (33) | 130 (34) | 686 (32) |
|  |  |  |  |  |  |
| Insulin within 91 days of diagnosis | Covid-19 | 136 (4) | 52 (12) | 45 (7) | 99 (3) |
|  | Controls | 92 (4) | 11 (7) | 19 (5) | 94 (4) |
|  |  |  |  |  |  |
| ‘Type 1 DM’ | Covid-19 | 60 (2) | 11 (3) | 21 (3) | 55 (2) |
|  | Controls | 56 (2) | 8 (5) | 14 (4) | 59 (3) |
|  |  |  |  |  |  |
| Male | Covid-19 | 1,405 (41) | 222 (53) | 308 (45) | 1,209 (37) |
|  | Controls | 940 (37) | 61 (37) | 128 (33) | 762 (35) |
|  |  |  |  |  |  |
| Current smoker | Covid-19 | 655 (19) | 78 (19) | 133 (20) | 615 (19) |
|  | Controls | 551 (22) | 31 (19) | 100 (26) | 480 (22) |
|  |  |  |  |  |  |
| Obese | Covid-19 | 1,502 (43) | 167 (40) | 256 (38) | 1,339 (41) |
|  | Controls | 990 (39) | 52 (31­) | 140 (36) | 761 (35) |
|  |  |  |  |  |  |
| ‘Asian’ ethnicity | Covid-19 | 542 (16) | 75 (18) | 98 (14) | 469 (14) |
|  | Controls | 282 (11) | 13 (8) | 38 (10) | 212 (10) |
|  |  |  |  |  |  |
| Insulin ever | Covid-19 | 181 (5) | 56 (13) | 46 (7) | 103 (3) |
|  | Controls | 113 (4) | 13 (8) | 22 (6) | 99 (5) |
|  |  |  |  |  |  |
| Oral hypoglycaemic drugs ever | Covid-19 | 1,145 (33) | 201 (48) | 245 (36) | 1,124 (35) |
|  | Controls | 784 (31) | 55 (33) | 122 (32) | 671 (31) |
|  |  |  |  |  |  |
| **Incident CVD patients (10,917)** | |  |  |  |  |
|  |  |  |  |  |  |
| Number | Covid-19 | 3,081 | 1,358 | 769 | 2,101 |
|  | Controls | 1,702 | 128 | 291 | 1,487 |
|  |  |  |  |  |  |
| Age (median, IQR, years) | Covid-19 | 61 (51 to 75) | 60 (50 to 72) | 59 (50 to 70) | 57 (46 to 70) |
|  | Controls | 60 (50 to 73) | 61 (51 to 74) | 58 (47 to 73) | 59 (49 to 74) |
|  |  |  |  |  |  |
| Male | Covid-19 | 1,575 (51) | 812 (60) | 435 (57) | 1,006 (48) |
|  | Controls | 838 (49) | 69 (54) | 140 (48) | 734 (49) |
|  |  |  |  |  |  |
| Current smoker | Covid-19 | 593 (19) | 220 (16) | 155 (20) | 453 (22) |
|  | Controls | 329 (19) | 31 (24) | 57 (20) | 365 (25) |
|  |  |  |  |  |  |
| Obese | Covid-19 | 936 (30) | 442 (33) | 251 (33) | 633 (30) |
|  | Controls | 429 (25) | 29 (23) | 53 (18) | 356 (24) |
|  |  |  |  |  |  |
| ‘Asian’ ethnicity | Covid-19 | 137 (4) | 70 (5) | 46 (6) | 109 (5) |
|  | Controls | 59 (3) | 0 (0) | 9 (3) | 47 (3) |
|  |  |  |  |  |  |
