# Supplementary material for: Cardiometabolic outcomes up to 12 months after COVID-19 infection. A matched cohort study in the UK
Source: PLoS Med. 2022 Jul 19;19(7):e1004052. doi: 10.1371/journal.pmed.1004052 (PMC9295991; doi:10.1371/journal.pmed.1004052)
Supplement: S7 Text — Figures are frequencies except where indicated. (DOCX) [file pmed.1004052.s010.docx]

| **Phase** | | **Case** | **Patient weeks** | **CVD Events** | **CVD incidence per 100,000 patient weeks (95% CI)** | | | **Diabetes diagnoses** | **DM incidence per 100,000 patient weeks (95% CI)** | | | |  |  |  |  |
| --- | --- | --- | --- | --- | --- | --- | --- | --- | --- | --- | --- | --- | --- | --- | --- | --- |
|  | |  |  | **Events** | **Rate** | **Lower** | **Upper** | **Events** | **Rate** | **Lower** | | **Upper** |  |  |  |  |
|  | |  |  |  |  |  |  |  |  |  | |  |  |  |  |  |
| **Before index date** | | Covid-19 | 14,367,449 | 1,123 | 7.82 | 7.37 | 8.29 | 1,794 | 12.49 | 11.92 | | 13.08 |  |  |  |  |
|  |  | Controls | 14,728,959 | 930 | 6.31 | 5.91 | 6.73 | 1,545 | 10.49 | 9.97 | | 11.03 |  |  |  |  |
|  | |  |  | | | |  |  |  |  |  |  | | |  |  |
| **Acute: up to 4 weeks from index** | | Covid-19 | 1,162,096 | 401 | 34.51 | 31.21 | 38.05 | 188 | 16.18 | 13.95 | | 18.66 |  |  |  |  |
|  |  | Controls | 1,149,284 | 86 | 7.48 | 5.99 | 9.24 | 115 | 10.01 | 8.26 | | 12.01 |  |  |  |  |
|  | |  |  | | | |  |  |  |  |  |  | | |  |  |
| **Post-acute: 5 to 12 weeks** | | Covid-19 | 2,300,296 | 293 | 12.74 | 11.32 | 14.28 | 385 | 16.74 | 15.11 | | 18.50 |  |  |  |  |
|  |  | Controls | 2,271,225 | 172 | 7.57 | 6.48 | 8.79 | 249 | 10.96 | 9.64 | | 12.41 |  |  |  |  |
|  | |  |  | | | |  |  |  |  |  |  | | |  |  |
| **Long: 13 to 52 weeks** | | Covid-19 | 11,014,384 | 962 | 8.73 | 8.19 | 9.30 | 1,852 | 16.81 | 16.06 | | 17.60 |  |  |  |  |
|  |  | Controls | 10,736,111 | 815 | 7.59 | 7.08 | 8.13 | 1,302 | 12.13 | 11.48 | | 12.80 |  |  |  |  |
|  | |  |  |  |  |  |  |  |  |  | |  |  |  |  |  |
