# Supplementary material for: Cardiometabolic outcomes up to 12 months after COVID-19 infection. A matched cohort study in the UK
Source: PLoS Med. 2022 Jul 19;19(7):e1004052. doi: 10.1371/journal.pmed.1004052 (PMC9295991; doi:10.1371/journal.pmed.1004052)
Supplement: S8 Text — Figures are coefficients (standard errors). (DOCX) [file pmed.1004052.s011.docx]

|  | **Model 1** | **Model 1 and consultation frequency** |
| --- | --- | --- |
|  |  |  |
| 1 to 4 weeks | 0.60 (0.09) | 0.59 (0.09) |
| 5 to 12 weeks | 0.24 (0.07) | 0.23 (0.07) |
| 13 to 52 weeks | 0.07 (0.04) | 0.07 (0.04) |
|  |  |  |

Model 1: adjusted for case, phase, case by phase interaction, gender, index month and index month squared
