# Supplementary material for: Cardiometabolic outcomes up to 12 months after COVID-19 infection. A matched cohort study in the UK
Source: PLoS Med. 2022 Jul 19;19(7):e1004052. doi: 10.1371/journal.pmed.1004052 (PMC9295991; doi:10.1371/journal.pmed.1004052)
Supplement: Attachment — Submitted filename: RezelPottsResponseToReviews28Feb2022.docx [file pmed.1004052.s012.docx]

**Cardiometabolic outcomes up to 12 months after COVID-19 infection. A matched cohort study in the UK**

***Editor’s comments:***

*Please add continuous line numbering throughout the manuscript to facilitate future reviews.*

Thank you, this has been added.

*Please update your title to ‘Cardiometabolic health outcomes after COVID-19 infection in the UK: A matched cohort study’, or similar.*

Thank you for the improved title, this has been added

*The Data Availability Statement (DAS) requires revision. Note that a study author cannot be the contact person for the data.*

This has been revised in the Editorial Manager pages.

*Please structure your abstract using the PLOS Medicine headings…*

The Abstract has been comprehensively revised as suggested.

*Citations should be in square brackets, and preceding punctuation.*

This has been corrected.

*At this stage, we ask that you include a short, non-technical Author Summary of your research to make findings accessible to a wide audience that includes both scientists and non-scientists. The Author Summary should immediately follow the Abstract in your revised manuscript. This text is subject to editorial change and should be distinct from the scientific abstract. Please see our author guidelines for more information:*

We now provide an author summary on page 3.

*Please ensure that the study is reported according to the STROBE guideline, and include the similar, to the Methods: "This study is reported as per the Strengthening the Reporting of Observational Studies in Epidemiology (STROBE) guideline (S1 Checklist)." When completing the checklist, please use section and paragraph numbers, rather than page numbers.*

We now provide a completed STROBE checklist.

*Please cite your protocol in the Methods section rather than the Acknowledgements.*

This has been corrected.

*Did your study have a prospective analysis plan? Please state this (either way) early in the Methods section.*

We now mention the study protocol and prospective analysis plan at the start of the Methods section, under ‘Ethical approval’ (page 6). The study protocol is provided as a supplementary document.

*Changes in the analysis-- including those made in response to peer review comments-- should be identified as such in the Methods section of the paper, with rationale.*

Thank you, this point is also mentioned by the statistical reviewer (Review 3). We now explain in the Methods section under ‘Analysis’ (page 8): ‘The prospective plan envisaged analysis in a time-to-event framework, with the date of Covid-19 as the start date. However, recognition of the importance of comparing risks from before the index date and adjusting for baseline values in a difference-in-difference analysis, prompted us to employ Poisson models to evaluate incidence of DM and CVD in periods before and after the Covid-19 diagnosis. However, time-to-event and log-linear models are generally equivalent and give similar results [27].’

*The terms gender and sex are not interchangeable*

We now refer to ‘sex’ throughout.

*Please indicate in the figure caption the meaning of the bars in Figure 1.*

We now indicate that these are confidence intervals (legend for Figure 1)

*Please refrain from including interpretation in the Results section (e.g. ‘The increase in CVD diagnoses was apparent in the last estimate for the pre-index period, which might imply that Covid-19 associated CVD might be recorded before Covid-19 diagnoses’ in paragraph 2 of the Results).*

This comment has now been omitted.

*Please provide titles and legends for all figures (including those in Supporting Information files).*

Titles and legends have been provided for all Figures.

*Please define any abbreviations used in Tables/Figures in the relevant legend.*

All abbreviations have now been defined.

*Please present the unadjusted incident rate ratios as well as the adjusted ratios in Table 3.*

Thank you, we have now added unadjusted estimates to Table 3. Unadjusted and adjusted estimates are very similar, we believe that this is because the difference-in-difference analysis already allows for baseline differences in risk before any covariate adjustment. We note (page 12) that ‘Covariate adjustment resulted in little change in the estimated incident rate ratios, with the difference-in-difference analysis already allowing for baseline differences in risk.’

*Please relocate the Ethical Approval information to the Methods section.*

This has been corrected.

*The Funding, Competing interests, Contributors, and Data sharing sections should be removed from the manuscript and captured as part of your submission form responses (in the event of publication, this information will be included as metadata).*

This has been corrected.

*Please ensure references are formatted per our guidelines*

The reference list has been formatted as requested.

*Please include [preprint] in reference 25.*

This has been added.

***Comments from the reviewers:***

***Reviewer #1:*** *It is essential to know the long-term cardiometabolic effects of COVID-19 so that measures can be taken early to prevent these after the acute phase of illness. In this context, this study's findings have important clinical implications.*

Thank you for this feedback.

1. *What type of SARS-CoV-2 variant was prevalent during the study period?*

Thank you for this point. We now add in the Introduction (page 4): ‘Since the first evidence of Covid-19 emerged in the beginning of 2020, there have been multiple waves of infection and multiple variants of SARS-CoV-2 have been identified.’

We also add in the Discussion (pages 17-18): ‘We also acknowledge that several variants of SARS-CoV-2 were dominant at different times during the course of the study (the original wildtype virus was superseded by the Alpha variant from December 2020 and the Delta variant from June 2021) and these variants might be associated with varying biomedical and clinical outcomes.’

*2.	Alcohol use, diet, physical activity, and lipid parameters (important risk factors for both CVD and diabetes) were not adjusted in the analysis. Are data not available? If yes, then acknowledge this as a limitation.*

Thank you, we now add under ‘Strengths and Limitations’ (page 17): ‘We also did not have sufficient data for alcohol use, diet, physical activity and lipid parameters which might have contributed as mediators of observed associations.’

2. *Cases and controls were matched on age, sex, and family practice, but the analyses were adjusted for age and sex. Isn't this over adjusting?*

Thank you, most authorities recommend adjusting for matching variables in order to reduce bias. We now add under limitations (page 18): ‘The study employed matching but we also adjusted for matching variables in order to avoid bias [38].’

3. *Apologies if I missed this - what % of patients were on glucose-lowering drugs, and what % were on insulin in the post COVID phase?*

Thank you, this information has now been provided in Supplementary Table 3.

4. *Comment on the % of participants with missing covariates.*

Thank you, we now add on page 10: ‘Covid-19 participants were more likely to have defined values recorded for covariates.’ We also elaborate on the issue of missing values further in the Discussion (page 17), as noted in responses to the other reviews.

We also explain in the Methods (page 9): ‘Analyses were adjusted for age, age-squared, sex, ethnicity, body mass index, systolic blood pressure, Charlson score, index month and index month squared. Missing values were represented by indicator variables (Table 1); we compared unadjusted and covariate adjusted estimates.’

***Reviewer #2:*** *The study is well executed, and statistical methods are sound.*

Thank you for this feedback.

*The authors describe some of the limitations but there are a few additional limitations that I will address below that the authors should address.*

1. *The matched control individuals are healthier than those that get COVID-19. Therefore, some of the higher incidence of DM after COVID-19 may be related to higher baseline risk for DM. Please address this in the discussion.*

Thank you for this comment. We now add under ‘Limitations’ (page 17): ‘Patients diagnosed with Covid-19 were generally less healthy than controls and this might, in part, account for differences in cardiometabolic outcomes. We employed a difference-in-difference analysis that allowed for baseline differences in risk, but there might have been residual confounding if there was misclassification of risk status from incomplete data recording.’

2. *Selection bias due to infection, severity of infection or availability for testing should be discussed.*

Thank you for this comment. We now address these points on page 16, where we now say: ‘. Covid-19 participants might predominantly include symptomatic cases and both symptomatic and asymptomatic contacts of cases who gave positive test results. We included patients with both confirmed and suspected Covid-19, but a sensitivity analysis found that restricting the analysis to PCR confirmed infections would not alter conclusions. However, PCR testing was associated with patient characteristics and reliance on PCR confirmation for participant selection might lead to bias. There might be a risk that the control group might be contaminated by undiagnosed or asymptomatic Covid-19 infections, this might be particularly so for the early stage of the pandemic when testing was not widely available. However, the Office for National Statistics Coronavirus (COVID-19) Infection Survey showed that at the height of the first wave of infection from 27 April and 10 May 2020, an average of 0.27% (95% confidence interval: 0.17% to 0.41%) of the general population had COVID-19 [36]. From October 2020 to October 2021, fewer than 2% of the population were infected with Covid-19 in any given week. Thus, the probability of a control participant having undiagnosed Covid-19 around the index date of a matched case is less than 0.02.’

*3. The authors have information on BMI and smoking only in a subset of the cohort (Table 1). To account for missingness they add a new variable for missing data. It is possible that missing data is masking a worse cardiometabolic health (like higher BMI), or increased smoking. The authors should perform sensitivity analysis in a sample without any missing data.*

Thank you for this comment. We agree that missing values represent a source of concern. However, a ‘complete case’ analysis can also yield biased results. We believe that this issue is best addressed through presentation of the unadjusted analyses, as suggested in the editorial comments. As noted earlier, covariate adjustment had minimal effect on the magnitude of estimates, while generally tending to provide greater precision. The similarity of adjusted and unadjusted estimates suggests that missing covariate values were unlikely to contribute any appreciable bias. We now comment on this under limitations (page 17): ‘The database enabled adjustment for a range of important covariates, but these were not always completely recorded. In health records, values are commonly missing ‘not at random’ making the application of imputation methods more difficult. However, estimates without covariate adjustment were of very similar magnitude to adjusted estimates, suggesting that there was no appreciable bias from missing covariate values.’

*Reviewer #3: Overall I think the study design and analysis is appropriate and the manuscript is clearly written.*

Thank you for this feedback.

*There are some differences from the planned analyses (in the protocol on CPRD) - in particular the use of Poisson regression instead of Cox models. What was the rationale for the change?*

Thank you for this comment. As noted above, we now explain in the ‘Analysis’ section (Methods) (page 8):

‘The prospective plan envisaged analysis in a time-to-event framework, with the date of Covid-19 as the start date. However, recognition of the importance of comparing risks from before the index date prompted us to employ Poisson models to evaluate incidence of DM and CVD in periods before and after the Covid-19 diagnosis date. However, time-to-event and log-linear models are generally equivalent and give similar results [27].’

*One covariate that I would consider important to consider as a confounder between COVID-19 and CVD/DM is healthcare utilisation. My experience is that DM diagnosis can be sensitive to increased use of medical care, i.e. more ill patients seek care and fasting glucose is commonly part of the standard panel of blood tests (and subsequent A1C testing). Are measures of healthcare utilisation able to be derived from the CPRD data? These could explain the more modest IRRs for DM in the post-acute and long phases. This is briefly acknowledged in the discussion, but some way of checking this would be helpful to put these findings into context.*

Thank you for this comment. We now note in the Results section (page 10): ‘Case patients had a median of nine consultations (interquartile range five to 18) after the index date, compared with six (two to 12) in controls.’ This higher frequency of consultations is consistent with the recent illness of case participants but, as noted by the reviewer, this could lead to more frequent opportunities for diabetes to be diagnosed. We now note under limitations (page 17): ‘We found that case participants had higher rates of family practice consultations, consistent with their recent illness. This increased medical surveillance could be associated with more frequent opportunities for diabetes to be diagnosed. However, illness from diabetes could itself lead to increased consultations.’ We adjusted analyses for consultation frequency (Supplementary Table 5 and page 13) but this had negligible effect on estimates.

*P2, Outcomes. Given that half of the sample were not exposed to Covid-19, i*

Apologies but transmission of this comment appears to have been incomplete.

*P4, Paragraph 2. Have there been any studies with longer-term follow-up of these type of samples?*

Thank you for this comment. We now add on page 5: ‘There is concern for the possible burden of ‘long Covid-19’ syndromes in patients recovering from Covid-19 but few studies have reported on long-term follow-up for large population-based samples. Al-Aly et al.[5] employed a data mining approach to Veterans’ Administration data and identified an increased burden of diverse health conditions between 30 days and six months after Covid-19. Knight et al.[20] suggested that arterial and venous complications remain elevated for 49 weeks after Covid-19. However, the timeline for recovery from Covid-19 remains poorly characterised.’

*P5, Paragraph 1. When establishing an exposure-outcome association, representativeness is not strictly necessary, but it would helpful to point to a reference or table that shows this. Does CPRD Aurum uniformly capture patients across England or are there regions that predominate? Not necessarily an issue - but I have seen previously studies with CPRD extracts that are more regionally focused (i.e. Northern England).*

Thank you for this comment, further information is given in reference 21. We now say (page 6): ‘The December 2021 release data, which cover approximately 20% of the population of England, are considered to be generally representative of the general population with regards to geographical distribution, deprivation, age and sex [21].’

*P5, Paragraph 2. How were the COVID-19 diagnoses linked to the CPRD data? Is this a routine linkage to patients from a notifiable diseases registry?*

Thank you, we now add (page 6): ‘Covid-19 test results are automatically transmitted from laboratories to patients’ family practices.’

*During this stage in England, were most COVID-19 diagnoses from PCR testing?*

Thank you, this information is provided in the Results section (page 9), where we say: ‘Covid-19 patients included 301,481 (80.9%) with clinical or laboratory confirmation of diagnosis on the index date, and 243,716 (65.4%) with a positive PCR test for SARS-CoV-2 on the index date. (Supplementary Table 1).’

*So just to clarify my understanding, effectively a pool of controls was created for each index date, including those not previously matched to a COVID-19 replacement (i.e. matched without replacement) and with no COVID-19 diagnosis before or on the index date? And stratified by home family practice?*

This is correct, thank you.

*Can the exclusions of patients with the numbers (e.g. excluding those with prevalent CVD/DM) be included in a flow diagram?*

Thank you, a flow diagram is now provided as Supplementary Figure 1.

*P5, Paragraph 3. From what sources do the CVD diagnoses come from? Patient self-report or linked hospitalisations?*

Thank you, we now add on page 6: ‘Coded records for CVD or DM derive from general practice consultations, hospital outpatient referrals and inpatient admissions.’

*Can you include a supplementary list (or reference) the drugs used to classify DM status? (i.e. ATC codes etc.).*

Thank you, code lists are now provided as a Supplementary file.

*Were you able to separate the drug use by indication - e.g identify where these were for Gestational DM, or metformin use for PCOS?*

Thank you, investigating small sub-groups of patients is beyond the scope of this study. We now comment (page 16): ‘While this study took a population perspective, clinical research is needed to determine whether sub-groups of patients at greater risk of hyperglycaemia, including those with pre-diabetes, pregnancy or polycystic ovary syndrome, are vulnerable to post-Covid-19 diabetes mellitus.’

*P6, Paragraph 2. Generally how up to date were these covariates?*

Thank you for this point, which we now acknowledge as a limitation (page 17): ‘Covariate values may change over time without being recorded at family practices. However, estimates without covariate adjustment were of very similar magnitude to adjusted estimates, suggesting that there was no appreciable bias from missing or misclassified covariate values.’

*Strictly speaking, the outcome in the study is CVD or DM, so the patients were matched on exposure status not on outcome status. I would consider adjusting the terminology (e.g. 'case and control patients') as this does give the impression that this is a case-control study with COVID-19 as the outcome.*

Thank you for this comment, we now refer throughout to ‘Covid-19 participants’ rather than ‘cases’.

*What was the rationale of examining glucocorticoids?*

Thank you for this comment, we now add on page 8: ‘Prescriptions for glucocorticoids were evaluated in patients diagnosed with DM because this therapy for severe Covid-19 is associated with diabetes risk [26].’

*P6, Paragraph 7 (and next page). The first two sentences of this paragraph (periods of 28 days, and calculations performed in days) seem to contradict each other. Or are they referring to separate parts of the analysis?*

Thank you, we now explain: ‘Person-time at risk was evaluated in terms of person-weeks of follow-up and incident events of CVD and DM were identified in each four-week period. Follow-up time was divided into periods of 28 days, from one year before the index date to one year after the index date.’ (page 8).

*Were the covariates in the analysis in the same form as in Table 1? Was age included as linear predictor? Was the fit of this checked?*

Thank you, we now explain on page 9: ‘Analyses were adjusted for age, age-squared..etc’

*What checks were made of the Poisson regression models - i.e. over-dispersion, distribution of residuals?*

Thank you for this comment. In the GLM approach, overdispersion may be a concern when Poisson models are fitted. However, when Generalised Estimating Equations are employed for estimation, the population-averaged model necessarily allows for overdispersion (as outlined here, for example, <http://support.sas.com/kb/22/630.html>). We note in the Methods (page 9): ‘The GEE model also allows for possible overdispersion. ‘

*How was missing data (i.e. covariates) managed in the analyses?*

Thank you, we now add (page 9): ‘Missing values were represented by indicator variables (Table 1).’

*Was the matched set the identity of each individual over the three time points? Or the pair of patients matched on age/sex/health centre?*

Thank you, we now refer to ‘the matched set of Covid-19 participant and control’

*Supp Table 2.*

*How was the overall difference estimated? Was this weighted according to follow-up time in each phase of the study?*

Thank you, the overall difference was estimated by including a factor to represent Covid-19 or control status, person-time provided the weighting. Our coding is now shown in new Supplementary Figure 2.

***Reviewer #4:*** *The relevance of the research question is unquestionable, and the results of this study (if correct) would be very important for public health.*

Thank you for this feedback.

*1. There is a high risk of information bias due to misclassification of the exposure (infection with SARS-CoV-2), particularly in the first wave. Access to testing was practically non-existent during the first months of the pandemic in the UK. Therefore, what is being mostly captured in electronic health records from primary care is symptomatic infection, rather than all infections with SARS-CoV-2. I acknowledge that the inclusion of 'suspected' cases in the analysis might have mitigated this effect to some extent, but the potential for controls to have been positive for SARS-CoV-2 during the first wave is non-negligible.*

Thank you for this point. We have addressed this by drawing on data from the Office for National Statistics Coronavirus (COVID-19) Infection Survey, which showed that even at the height of the first wave only 0.27% of the general population were found to have Covid-19 in a two-week period. We now add under limitations (page 16):

‘There might be a risk that the control group might be contaminated by undiagnosed or asymptomatic Covid-19 infections, this might be particularly so for the early stage of the pandemic when testing was not widely available. However, the Office for National Statistics Coronavirus (COVID-19) Infection Survey showed that at the height of the first wave of infection from 27 April and 10 May 2020, an average of 0.27% (95% confidence interval: 0.17% to 0.41%) of the general population had COVID-19. From October 2020 to October 2021, fewer than 2% of the population were infected with Covid-19 in any given week. Thus, the probability of a control participant having undiagnosed Covid-19 around the index date of a matched case is less than 0.02.’

*2.	The authors state that they conducted a matched cohort study, but the results included two components:*

Thank you for this comment. We would like to affirm that the analysis is based on a single matched cohort study. The analysis of person time from before the index date is based on this same matched cohort and all participants with prevalent diabetes and CVD from one year before the index date were excluded. Thus, there is a single analysis and not two separate components.

*2.1.	In the first component, the authors describe the incidence of outcomes prior to the index date in the exposed and unexposed cohorts. There is vast evidence that people with comorbidities have higher risk of severe COVID-19 outcomes, should they get infected. As people with comorbidities are more likely to have more severe disease, they will also be more likely to present in primary care with symptoms and have the disease recorded in their health record. For this reason, I strongly disagree with the authors' claim in the discussion: "The study showed that patients recorded with Covid-19 in primary care are at slightly greater risk of CVD and DM even before the Covid-19 diagnosis." This study showed that people with history of CVD and DM are more likely to present with symptomatic infection of SARS-CoV-2, which we already knew.*

Thank you for this comment. However, we would like to emphasise that patients with pre-existing diabetes or cardiovascular disease were excluded from the analysis entirely. Consequently, we disagree that the study might show that people with history of CVD and DM are more likely to present with symptomatic infection of SARS-CoV-2. We now note this point in the Methods section under ‘participant selection’ (page 7) where it now reads ‘Matched sets that included case or control patients with prevalent CVD or DM, diagnosed within 12 months of the start of record or more than 12 months before the index date, were excluded.’

We also note that all 372,816 ‘case’ patients were diagnosed with Covid-19 as compared to none of the controls. In the 12 months before the index date 2,177 case patients and 1,311 controls were diagnosed with CVD and 2,612 case patients and 1,970 controls were diagnosed with diabetes (as shown in Table 2). This is the basis for the statement (page 13, last line) that ‘The study showed that patients recorded with Covid-19 in primary care are at slightly greater risk of CVD and DM even before the Covid-19 diagnosis.’

To explain this we now add on page 14: ‘While patients with pre-existing DM or CVD may be at greater risk of symptomatic Covid-19, we excluded all patients with DM or CVD as pre-existing conditions at study entry.’

*In addition, there is also a survival bias inherent in this analysis: the authors described the incidence of CVD and diabetes in two cohorts of people who survived long enough to present with symptomatic COVID. People who died from CVD are not included in the denominator.*

Thank you for this point. We now also add on page 14: ‘Patients who had already died from CVD or DM were necessarily excluded also.’

2.2. *I am also concerned with the execution of the second component of the study (i.e. the association between COVID-19 and incident CVD and DM). The authors stated that: "Matched sets that included case or control patients with prevalent CVD or DM, diagnosed within 12 months of the start of record or more than 12 months before the index date, were excluded." This is problematic. Patients with prevalent disease should have been excluded before matching, to avoid excluding pairs where the chosen control had CVD/DM history, as another control could have been selected instead of dropping the case.*

Thank you for this point. Controls were selected from a complete list of patients included in CPRD Aurum. This list only includes details of year of birth, sex and start and end of registration. CVD and DM status could only be determined after controls were selected and their full data was extracted from the database. For this study, we could only include matched sets in which neither cases nor controls already had DM or CVD. We now explain this point on page 7, where it now reads: ‘Since it was not possible to determine whether control participants had CVD or DM until after controls were selected and data extracted, matched sets that included either case or control patients with prevalent CVD or DM, diagnosed within 12 months of the start of record or more than 12 months before the index date, were excluded from analysis.’

2.3. *Also, the precise definition of the outcomes is not given (perhaps these could be included in appendix?)*

Thank you, we now provide lists of codes used to define relevant conditions as a supplementary file.

2.4. *It is not clear to me what counted as an 'incident' event. For example, people with a DM diagnosis 5 years prior to COVID - were they excluded from the denominator in the descriptive 'incidence' analysis and dropped from the matched cohort study? This is unclear.*

Thank you for this comment, we have now adjusted the wording on page 7, so that it now reads: ‘The study outcomes were first ever recorded CVD and DM diagnoses.’ As noted above, all patients with prevalent (or pre-existing) DM and CVD were excluded from the analysis.

*3.	Following from the previous comment, could the authors provide the precise definitions of all variables used in this study and a flowchart for the patients' inclusion/exclusion. Some variables included in the models are prone to missingness but there is no mention to missing data in the methods. In line with best practice in the field, and to ensure transparency and reproducibility, could the authors make all code lists and patient selection information available in appendix?*

Thank you for this comment. We now provide a flow chart that details participant selection as Supplementary Figure 1. We also provide a supplementary file that documents the case definitions for Covid-19 infection, cardiovascular disease and diabetes mellitus.

*4.	The authors used 'long-COVID' to refer to patients who had SARS-CoV-2 infection 13 to 52 weeks after the index date. I acknowledge that this is clearly defined in the paper but 'long-COVID' has been used since the beginning of the pandemic to describe the state where symptoms linger after SARS-CoV-2 infection. I kept having to remind myself that 'long-COVID' in this paper was defined by time since index. Wondered if simply listing the time since index throughout the paper would make the manuscript clearer.*

Thank you for this suggestion. We now refer throughout to the periods ‘from five to 12 weeks after Covid-19 diagnosis’ and from 13 to 52 weeks after Covid-19 diagnosis’.

*I have other minor comments:*

*Abstract*

*The abstract needs revision as it contains several typos. COVID-19 and SARS-CoV-2 infection are used interchangeably in the abstract - could this be corrected as the two are not equivalent. As mentioned above, I would also avoid the term 'Long covid'. The section for 'Outcomes' also includes the methods description - perhaps a mistake?*

Thank you for these comments. The Abstract has now been comprehensively revised. We generally refer to Covid-19 rather than SARS-CoV-2 infection. We now avoid referring to ‘long COVID-19’. The sub-headings for the Abstract have been revised, consistent with the Journal requirements, as outlined above.

*Introduction*

*The introduction gives a good rationale for this study. However, some sentences could be revised to improve the coherency of the argument being made:*

(1) *"Acute Covid-19 infection has been associated with new-onset cardiovascular disease (CVD) and diabetes mellitus (DM.)" Could you add a reference for this claim? This sounds like a fact and one wonders about what this study adds to the current body of research.*

Thank you, we now cite new reference [5] and comment (page 4): ‘While acute Covid-19 infection has been associated with new-onset cardiovascular disease (CVD) and diabetes mellitus (DM), [5] longer-term outcomes up to one year following the infection have not been well-characterised.’

(2) *"New onset hyperglycaemia has been reported in Covid-19 patients and is associated with worse prognosis" Please add a reference.*

Thank you, we now say (page 4): ‘New onset hyperglycaemia has been reported in Covid-19 patients and is associated with worse prognosis [5, 11].’

(3) *"There were in excess of 1,000 deaths per day in the UK during April 2020 and January 2021" This is incorrect. According to the reference given by the authors, between April 2020 and January 2021, there were fewer than 1000 deaths registered in most of the days.*

Thank you, we have now clarified the wording by saying (page 4): ‘There were in excess of 1,000 deaths per day in the UK at the peak of the first wave in April 2020 and the peak of the second wave in January 2021’

(4) *"However, susceptibility to and severity of Covid-19 infection is known to be associated with cardiometabolic risk." What does susceptibility mean in this sentence? Susceptibility to the infection?*

Thank you, we now say (page 5): ‘However, susceptibility to Covid-19 and the severity of illness are known to be associated with cardiometabolic risk.’

*(5)	"We employed the Clinical Practice Research Datalink (CPRD), a national database of anonymised primary care electronic health records, to identify a cohort of Covid-19 patients, comparing new CVD and DM diagnoses to a matched cohort with no Covid-19 diagnosis." Please note that none of the primary care databases at CPRD has national UK coverage. CPRD GOLD is no longer representative of the UK population in terms of geographical distribution of contributing practices. CPRD Aurum includes data from GP practices in England and Northern Ireland, and do not include practices from the devolved nations.*

Thank you, we now omit the word ‘national’ and adjust the wording so that it now reads (page 5): ‘We employed the Clinical Practice Research Datalink (CPRD), a database of anonymised primary care electronic health records, to identify a cohort of Covid-19 exposed patients in comparison with a matched cohort with no Covid-19 diagnosis.’

*Methods*

1. *The number of practices varies across builds of the CPRD Aurum primary care database. Please clarify which build was used before stating the number of practices included in the database.*

Thank you, we agree that the CPRD represents an open cohort, with the precise number of registered patients changing daily. We now refer to the December 2021 release (page 6): ‘The CPRD Aurum is a large database which includes comprehensive medical record data for a current total of 1,366 family practices in England with approximately 13.4 million currently registered patients in the December 2021 release.’

2. *Please cross-check the number of patients registered in practices actively contributing with data to CPRD Aurum in the build used for this study. The % of the population covered is probably outdate too. I suggest that the author look for these numbers in the CPRD Release notes for the specific build used in this study.*

Thank you, we now say (page 6): ‘The December 2021 release data, which cover approximately 20% of the population of England…’

3. *Minor: please delete '18.5' prior to 'Kg/m2' in all categories.*

Thank you, this has now been corrected.

4. *Could the authors also include a DAG or some justification as to why they considered those variables as potential confounders of the association between COVID-19 and incident CVD / DM outcomes?*

Thank you, we now address this by saying (page 8): ‘Covariates selected because of known associations with cardiovascular disease and diabetes mellitus included smoking status etc’ and ‘We also employed the Charlson index to summarize comorbidities that may be associated with the severity of illness in Covid-19 [22].’

5. *The information on PCR testing is interesting. Could the authors clarify how did they ascertain who got a PCR positive test?*

Thank you, we now explain (page 7): ‘We conducted a sensitivity analysis including only patients with a medical code recorded for polymerase chain reaction (PCR) test confirmed Covid-19.’ As noted in an earlier response, testing laboratories automatically transfer patients’ test results to their general practices.

*6.	What were mortality data used for?*

Thank you for this comment. We agree that the mortality data were not directly relevant and have now been omitted from Table 2.
