# Supplementary material for: Cardiometabolic outcomes up to 12 months after COVID-19 infection. A matched cohort study in the UK
Source: PLoS Med. 2022 Jul 19;19(7):e1004052. doi: 10.1371/journal.pmed.1004052 (PMC9295991; doi:10.1371/journal.pmed.1004052)
Supplement: Attachment — Submitted filename: RezelPottsResponseToReviews20April2022.docx [file pmed.1004052.s013.docx]

**Cardiometabolic outcomes up to 12 months after COVID-19 infection. A matched cohort study in the UK**

**Requests from the editors:**

*Please provide an explanation as to why your use of the same data system is different from Rev #4.*

Thank you for this comment. We have now responded to Reviewer 4 comments by sampling a new set of control participants using the procedure suggested.

We also took this opportunity to update the analysis of data for Covid-19 cases and control participants to the March 2022 release of CPRD Aurum. This provides follow-up to the end of January 2022, as compared to end of October 2021 for data in the December 2021 release that was employed previously. This updating ensures that most participants now have 12 months complete follow-up and additional person-time has now accrued for longer durations of follow-up.

We would like to emphasise that the new version of the paper employs the same analytic code that was employed previously, benefitting from the previous review comments. The numerical data in the paper and supplement have now been updated with all changes highlighted.

While the main findings of the study remain unchanged, there is a difference in interpretation for the incidence of diabetes at longer durations of follow-up, as noted below. The study now provides evidence that the incidence of diabetes returns to baseline during the long Covid-19 period. We now provide additional secondary analysis to estimate adjusted rate ratios by four- week period. These results provide evidence that the incidence of diabetes might remain elevated up to 20-23 weeks after Covid-19 but not after this. However, our headline conclusions are based on the primary analysis reported previously.

All changes to the paper have been highlighted.

Thank you for this opportunity to revise and update the paper. We believe that this revised version of the paper now provides the most up-to-date information available concerning this emerging topic of concern.

**Comments from the reviewers:**

*Reviewer #1: Thank you authors for addressing my comments and revising the manuscript accordingly. Please change "moderators" to "confounders" in your response to comment 2.*

Thank you, this change has now been made. (page 18, line 376)

*Reviewer #2: The authors have sufficiently addressed all my comments.*

Thank you for this feedback.

*Reviewer #3: Thanks for the revised manuscript and response to my original queries. Overall, the revisions and responses clarified all of my original review and apart from one very small change I recommend that the manuscript be accepted for publication. The editor can safely ignore the cut-off comment - this should have been removed before submitting the initial review.*

*The explanation for the change in analysis to allow for a difference-in-difference analysis makes sense to me, and I agree that generally you will see similarity between HRs and IRRs when each analysis is applied to the same data. The DID approach with this type of data really looks effective.*

*I agree with your reply to Reviewer 1, point 2 about the adjustment for age and sex. This is an approach often recommended as being 'doubly-robust' in that if one (e.g. matching) fails then the other (e.g. covariate) will be effective. Even if data is already well-matched and confounding with the exposure is 'eliminated', it is still a good idea if there is an association between these covariates and the outcome, as it shrinks the unexplained variance and you end up with more precise estimates of standard error (and hence narrower 95% CI) of other estimates in the model.*

Thank you for feedback on these points of agreement.

*The one small pedantic change requested - for categories of BMI (L114), I would use "18.5 - <25 kg/m2", as if it were "18.5-24.9" strictly speaking someone with a BMI of 24.95 would not be classified into any category.*

Thank you, this suggestion has now been incorporated. (page 8, lines 114-115)

**Reviewer #4: RE Comment 2.2:**

*Thank you for clarifying what was done. The authors stated that "CVD and DM status could only be determined after controls were selected and their full data was extracted from the database". This is untrue. The 'define' tool, in the CPRD data platform, allows researchers to obtain a list of all patients with a record of a given condition (as defined in the code list). This includes CVD and DM. Once you ran the define and obtain a list of patients with history of CVD and DM, this can be merged with the denominator files (the complete list of patients that you mentioned) to identify and exclude those that are not eligible for the study. Matching should only be done after creating the pool of potentially eligible controls. I understand this is not what was done, and I acknowledge that the authors were transparent about this in the revised version of the manuscript. I do not know if the non-standard design of the study introduced bias, and if yes, of what magnitude. I suspect this won't change the study conclusion, but I do struggle with this error that could be easily avoided. A statistical reviewer may be better suited to advise. Thank you for clarifying all other points and thank you for the opportunity to review this paper.*

Thank you for these comments. We have now adopted the suggestion to sample control participants after incorporating the CVD/DM diagnosis date obtained using the Define tool.

We now add on page 7, lines 90-94: ‘Patients were excluded from eligibility as controls if their record ended before the matched case index date, as were patients with prevalent CVD or DM recorded more than 12 months before the index date or within one year of the start of their record.’

A small number of controls was subsequently excluded with prevalent CVD or DM identified from their full medical records (Supplementary Figure 1).

As noted above, we also updated the data extract to the March 2022 release of CPRD Aurum to provide more complete follow-up. Changes to the paper may be explained both by the new sample of controls and the updated data extract, with the latter providing more complete data for longer durations of follow-up.

In this updated version, the main findings are generally consistent with those reported previously. However, the new dataset provides evidence that the incidence of DM is not increased overall from 13 to 52 weeks after Covid-19. We note this as follows:

Abstract (page 2): ‘DM incidence remains elevated up to 12 weeks following Covid-19 before declining.’

Author Summary (page 3): ‘The risk of new diabetes mellitus remains increased for at least 12 weeks following Covid-19 before declining.’

Results (page 12, line 220): ‘In the period from 13 to 52 weeks after diagnosis, the adjusted incidence rate ratio for CVD was 0.80 (0.73 to 0.88) while for DM this was 1.07 (0.99 to 1.16).’

Discussion (page 14, line 284): ‘There was no evidence overall that Covid-19 was associated with any additional increase in DM and CVD events from 13 to 52 weeks, above that observed in controls. However, secondary analysis by four-week period provided evidence that DM events might not return to baseline until 20 to 23 weeks after Covid-19.’

This last comment refers to a new secondary analysis, in which adjusted rate ratios were estimated separately for each four-week period following Covid-19 onset. We now add:

Methods (page 9, line 151): ‘In secondary analyses, we estimated adjusted rate ratios separately for each four-week period following Covid-19 diagnosis, using the same analytical approach.’

Results (page 12, line 236): ‘When adjusted rate ratios were estimated by four-week period (Figure 3 and Supplementary Table 6), there was no evidence that CVD events might be increased beyond eight to 11 weeks after Covid-19 onset (RR 1.15, 95% confidence interval 0.94 to 1.42, P=0.18). There was evidence of a net reduction in CVD events, compared with baseline and controls, from weeks 20 to 23 onwards. There was evidence that DM incidence might be increased up to 20 to 23 weeks after Covid-19 onset (1.29, 1.08 to 1.55, P=0.005) but not from 24 to 27 weeks onwards (1.03,	0.86 to 1.22, P=0.78).‘

Thus, we conclude (Abstract): ‘People without pre-existing CVD or DM who suffer from COVID-19 do not appear to have a long-term increase in incidence of these conditions.’
