# Supplementary material for: Cardiometabolic outcomes up to 12 months after COVID-19 infection. A matched cohort study in the UK
Source: PLoS Med. 2022 Jul 19;19(7):e1004052. doi: 10.1371/journal.pmed.1004052 (PMC9295991; doi:10.1371/journal.pmed.1004052)
Supplement: Attachment — Submitted filename: RezelPottsResponseToReviews19May2022.docx [file pmed.1004052.s014.docx]

**Cardiometabolic outcomes up to 12 months after COVID-19 infection. A matched cohort study in the UK**

**Requests from the editors:**

*Please clarify your methodology in response to the comments of reviewer #4.*

Thank you, please see our response below to Reviewer 4 comments.

*Please use track changes rather than highlighting to indicate changes in your marked up manuscript – the previous version provided did not highlight all the changes made between the R2 and R3 versions.*

Thank you, all changes to this revision have now been tracked.

*Please ensure all figures can be interpreted without the need to refer to the main text (Figure 1 for example needs either a key or information in the legend to define which line represents patients vs controls).*

Thank you, we have now adjusted Figure 1 legend (line 576) to read ‘Figure 1: Incidence rates for diabetes mellitus and cardiovascular diseases (per 100,000 patient weeks) for Covid-19 patients (red) and controls (blue) for 4-week periods. Bars are 95% confidence intervals.’ We believe the other Figure legends are complete.

*Please add the following statement, or similar, to the Methods: ""This study is reported as per the Strengthening the Reporting of Observational Studies in Epidemiology (STROBE) guideline (S1 Checklist)."*

Thank you, this sentence has now been added to the Methods section at line 67.

*Changes in the analysis—including those made in response to peer review comments—should be identified as such in the Methods section of the paper, with rationale.*

Thank you, we now add at line 160: ‘We recognised that incidence of CVD and DM might change during the period from 13 to 52 weeks after Covid-19 diagnosis. Therefore, in secondary analyses, we estimated adjusted rate ratios and their confidence intervals for the comparison of each four-week period following Covid-19 diagnosis with baseline, using the same analytical approach.’

We also add at line 169: ‘Following peer-review, we included a sensitivity analysis to evaluate whether adjusting for consultation frequency might explain association of Covid-19 with diabetes incidence.’

*Please ensure all supplementary tables are cited in the main text (I couldn’t locate a reference to Supplementary Table 2).*

Thank you, Supplementary Table 2 (now S4 Text) is now cited in the Results section at line 236. The remaining supplementary materials have now been renumbered according to the formatting guidelines and are submitted as separate files.

**Reviewer #3:**

*I noted after the last review that excluding the matched sets for a members with prevalent DM/CVD shouldn't introduce bias, it's reassuring to see that when this step is applied earlier there is no appreciable difference in the pattern of DM incidence. The additional analysis by 4-week period with the extended data is quite interesting - better not to speculate about mechanisms, but I will just add that I think this is a valuable addition.*

Thank you for this feedback.

*One very small check, and an addition as well. For fig 3 the confidence intervals come from the SE estimates of the main model? If so then I would just then specify the method used to create the smoothed line.*

Thank you, we now re-word the explanation of the analysis and plotting method at line 162, where we now say: ‘in secondary analyses, we estimated adjusted rate ratios and their confidence intervals for the comparison of each four-week period following Covid-19 diagnosis with baseline, using the same analytical approach. Estimates were plotted with a smoothed curve fitted using the loess method.’

*Great paper - whoever was doing the analysis might need a day off after managing to update the paper with additional data so quickly*

Thank you for this feedback and recommendation.

**Reviewer #4:**

*Thank you for addressing my comments. Unfortunately, I don't think the methods are clear. The authors described using the Feb 2021 version of Aurum to identify patients with COVID-19. They then mention that controls (I am assuming they meant to say controls when they said 'patients') 'were randomly selected from the registered population of CPRD Aurum March 2021 release' (p. 7, lines 88-89). Subsequently, the authors mentioned that 'data were updated to the March 2022 release of Aurum for final analysis'. I am unclear why three different versions of CPRD Aurum were used, when all information you need is provided in the most recent version of the database. This gives me no confidence that the authors handled the data properly, and therefore I cannot recommend this paper for publication.*

Thank you for these comments.

We now explain in the Methods section at line 76: ‘The database includes data for all registered patients at participating general practices, except for a negligible number of patients who opt-out of data collection. Each patient has a unique anonymised numerical identifier that remains the same at each update of the database. Patient records may therefore be tracked through successive releases of the database which is updated monthly.’

At line 87, we now explain: ‘At the inception of the study, data for all patients with a diagnosis of Covid-19 were extracted from the February 2021 release of CPRD Aurum.’

At line 94, we now explain: ‘Control patients were randomly sampled from the registered population of CPRD Aurum March 2021 release which provided the most up-to-date information on the database at the time of sampling.’

At line 101, we now explain: ‘Using the unique identifiers for patients in the sample, data were updated to the March 2022 release of CPRD Aurum for final analyses, providing follow-up to 31^st^ January 2022.’

This approach was determined by the need to have the most up-to-date information for the existing sample at the time of the final analysis. We did not believe that it would be appropriate to draw a new sample of patients with the manuscript having already been peer-reviewed.
